# Extract of Corpus Lutem in Disturbances of Artificial and Physiologic Menopause

**Published:** 1910-01

**Authors:** 


### ABSTRACT.

### Extract of Corpus Lutem in Disturbances of Artificial and Physiologic Menopause.

Morley, in the November number of the *Journal of the Michigan State Medical Society,* reports his results in eighteen cases. This report is a continuation of the one that appeared in the August number of the *Detroit Medical Journal.* The author used an extract made from the corpora lutea of beef ovaries rather than an extract of the entire ovary as the consensus of opinion seems to be that the internal secretion of the ovary is produced by the yellow body. The extract is given in five grain doses three times a day, one-half to one hour before meals. His results in eighteen cases may be summed up as follows:

Five were cured, twelve were improved and one obtained no relief. Included in the twelve cases that were improved are grouped those, that are still taking the extract. A permanent cure may result in a few of the cases under treatment. Of the eighteen cases, fourteen suffered from disturbances of operative or artificial and four from these of natural or physiologic menopause. While the results obtained in so small a group of cases do not warrant the drawing of any definite conclusions, still the author thinks that the results are favorable enough to justify a continuance of the treatment in other cases, where there is a disturbance incident to artificial or physiologic menopause.

To differentiate a tender spot from a simulated pain, it will often be observed that pressure on the former causes a decided increase of pulse rate, while in simulation it does not.—*American Journal of Surgery.*

